# Systolic blood pressure variability in late-life predicts cognitive trajectory and risk of Alzheimer’s disease

**DOI:** 10.3389/fnagi.2024.1448034

**Published:** 2024-10-03

**Authors:** Xiao-Lu Li, Ruo-Tong Wang, Chen-Chen Tan, Lan Tan, Wei Xu

**Affiliations:** ^1^Department of Neurology, Qingdao Municipal Hospital, Qingdao University, Qingdao, China; ^2^Medical College, Qingdao University, Qingdao, China; ^3^Department of Neurology, Qingdao Municipal Hospital, Dalian Medical University, Dalian, China

**Keywords:** Alzheimer’s disease, systolic blood pressure variability, amyloid, brain metabolism, white matter hyperintensities

## Abstract

**Background:**

The relationship of systolic blood pressure variability (SBPV) with Alzheimer’s disease (AD) remains controversial. We aimed to explore the roles of SBPV in predicting AD incidence and to test the pathways that mediated the relationship of SBPV with cognitive functions.

**Methods:**

Longitudinal data across 96 months (T_0_ to T_4_) were derived from the Alzheimer’s disease Neuroimaging Initiative cohort. SBPV for each participant was calculated based on the four measurements of SBP across 24 months (T_0_ to T_3_). At T_3_, logistic regression models were used to test the SBPV difference between 86 new-onset AD and 743 controls. Linear regression models were used to test the associations of SBPV with cognition and AD imaging endophenotypes for 743 non-demented participants (median age = 77.0, female = 42%). Causal mediation analyses were conducted to explore the effects of imaging endophenotypes in mediating the relationships of SBPV with cognitive function. Finally, Cox proportional hazard model was utilized to explore the association of SBPV with incident risk of AD (T_3_ to T_4_, mean follow-up = 3.5 years).

**Results:**

Participants with new-onset AD at T_3_ had significantly higher SBPV compared to their controls (*p* = 0.018). Higher SBPV was associated with lower scores of cognitive function (*p* = 0.005 for general cognition, *p* = 0.029 for memory, and *p* = 0.016 for executive function), higher cerebral burden of amyloid deposition by AV45 PET (*p* = 0.044), lower brain metabolism by FDG PET (*p* = 0.052), and higher burden of white matter hyperintensities (WMH) (*p* = 0.012). Amyloid pathology, brain metabolism, and WMH partially (ranging from 17.44% to 36.10%) mediated the associations of SBPV with cognition. Higher SBPV was significantly associated with elevated risk of developing AD (hazard ratio = 1.29, 95% confidence interval = 1.07 to 1.57, *p* = 0.008).

**Conclusion:**

These findings supported that maintaining stable SBP in late life helped lower the risk of AD, partially by modulating amyloid pathology, cerebral metabolism, and cerebrovascular health.

## Introduction

Alzheimer’s disease (AD) is the most common form of dementia and one of the principal causes of physical disability, institutionalization, and decreased quality of life among the elderly (Hodson, 2018; Qiu et al., 2009). Amyloid pathology (Jack et al., 2016), brain metabolism (Ou et al., 2019), and vascular health (Mortamais et al., 2014) have been identified as contributing factors to AD. It was emphasized that effective interventions in pre-existing diseases and lifestyle may be promising options for preventative strategies (Xu et al., 2015; Yu et al., 2020; Alzheimers Association Report, 2022). Late-life systolic blood pressure (SBP) was revealed as an important predictor of developing AD (Ou et al., 2020; Li et al., 2007; Qiu et al., 2003). However, the relationships of SBP with AD are conflicting (Ruitenberg et al., 2001; Morris et al., 2001), which might be partially due to the underestimation of BP dynamics across the life-span. Measurements of SBP variability (SBPV), as a dynamic feature of SBP, could thus help lower the bias (de Heus et al., 2019; de Heus et al., 2021). SBPV indicates the degree to which an individual’s blood pressure fluctuates over time. It was revealed as an important risk factor for target organ damage, independent of SBP levels. For example, it was suggested that higher SBPV was associated with elevated risk of cardiovascular events (Stevens et al., 2016) and subclinical brain disease (Ma et al., 2020a; Ma et al., 2020b). These studies illustrate the importance of SBPV for brain health. Recently, it was indicated that higher SBPV was associated with elevated risk of cognitive decline or all-cause dementia (Alperovitch et al., 2014; Rouch et al., 2020; Mahinrad et al., 2023; Ernst et al., 2021; den Brok et al., 2023), though the conclusion remained controversial (van Middelaar et al., 2018). Till now, little is known about whether late-life SBPV could predict AD dementia or its biomarkers. Moreover, the underlying biological mechanisms by which SBPV affects cognition and AD is unclear.

Herein, using longitudinal data from the Alzheimer’s Disease Neuroimaging Initiative (ADNI) cohort, we firstly aimed to explore the relationship of SBPV with AD risk by a) depicting the SBP trajectory before AD diagnosis and comparing the difference of SBPV between incident AD patients and their counterparts, and b) testing the roles of SBPV in predicting incident AD among non-demented elders. Next, we aimed to verify a prior hypothesis that SBPV could influence cognition by modulating amyloid pathology, brain metabolism, and vascular health.

## Materials and methods

### Participants

Data was derived from the Alzheimer’s Disease Neuroimaging Initiative (ADNI) study^1^ (Petersen et al., 2010; Weiner et al., 2010). The primary objective of ADNI is to create positron emission tomography (PET), magnetic resonance imaging (MRI), genetic, and biochemical markers which can be used to detect and monitor AD at an early stage. Volunteers with normal cognition (NC), mild cognitive impairment (MCI), or mild AD dementia were continuously recruited from multiple centers throughout North America. At entry, each participant underwent a detailed physical examination, a comprehensive neuropsychological evaluation, and an in-person interview to obtain baseline information. Follow-up measurements were repeated at 12-month intervals (Weiner et al., 2010). Further insights can be obtained by visiting https://adni.loni.usc.edu/data-samples/adni-data/. ADNI was approved by institutional review boards of all participating institutions. Written informed consent was obtained from all participants or authorized representatives according to the 1975 Declaration of Helsinki.

**FIGURE 1:**
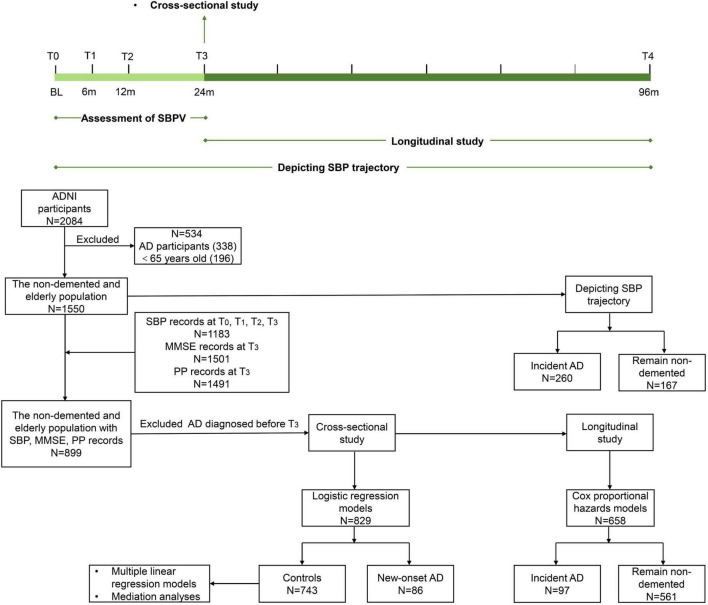
Timeline of study design and flowchart for the overall selection process. SBPV was defined as the standard deviation of systolic blood pressure at T_0_, T_1_, T_2_, and T_3_ and divided into quartiles. SBP trajectory was based on 96-month follow-up. Cross-sectional studies were conducted at T_3_. Longitudinal studies were conducted from T_3_ onwards. Participants who were diagnosed with AD and aged <65 years old at baseline (T_0_) were excluded from 2084 ADNI participants. A total of 1550 non-demented and elderly participants were followed up for 96 months (T_0_ to T_4_) to depict the SBP trajectory. A total of 899 non-demented and elderly participants with records of SBP, PP, and MMSE were selected. After excluding incident AD before T_3_, 829 were included for logistic regression models. A total of 743 non-demented participants were included for multiple linear regression models and mediation analyses. For the longitudinal study, Cox proportional hazards models were applied to 658 participants.

In the present study, three steps were conducted to explore the association of SBPV in late life with cognitive trajectory and risk of AD. First, data of 2,084 ADNI participants were downloaded and those diagnosed with AD and aged <65 years old at baseline (named T_0_) were excluded. Then, 1550 non-demented and elderly participants were followed up for 96 months (T_0_ to T_4_). To depict the SBP trajectory before AD diagnosis, participants were categorized into two groups: participants who were diagnosed with AD (N = 260) during the follow-up and those who remained non-demented (N = 167) over the 96 months. Second, 899 non-demented and elderly participants with records of SBP at T_0_, T_1_, T_2_, and T_3_, PP and MMSE records at T_3_ were included for cross-sectional analyses, after excluding participants who were diagnosed with AD before T_3_: (1) logistic regression models were conducted between 86 participants with new-onset AD at T_3_ and 743 controls who were free of dementia at T_3_. (2) Multiple linear regression models and mediation analyses were conducted in 743 non-demented participants. Third, for longitudinal analyses, participants who were free of dementia at T_3_ were followed up for 72 months (T_3_ to T_4_). Subsequently, Cox proportional hazards model was applied for 658 participants, including 97 with incident AD and 561 who remained non-demented (Figure 1).

### Blood pressure measurements

Blood pressures were measured at baseline and at each follow-up. Participants were instructed to remain calm and avoid talking during and shortly before blood pressure measurement. Blood pressure was taken from the same arm, at a similar time of day, by the same person, using the same device and cuff, whenever possible. SBPV was defined as the within-individual standard deviation (SD) of four SBP measurements at T_0_, T_1_, T_2_, and T_3_ (Figure 1). SBPV was divided into four quartiles (Q1 to Q4). Pulse pressure (PP) was calculated as systolic minus diastolic blood pressure.

### Cognitive assessments

The general cognitive function was evaluated by the Alzheimer’s Disease Assessment Scale-13 item cognitive subscale (ADAS-13), which is a test battery assessing memory function, reasoning, language function, orientation, and praxis. Composite scores for memory (MEM) and executive function (EF) were derived using data from the ADNI neuropsychological battery via item response theory methods. With the exception of ADAS-13, higher scores were indicative of enhanced cognitive performance in all neuropsychological assessments. The composite scores have been validated previously (Crane et al., 2012; Gibbons et al., 2012).

### AD diagnosis

AD diagnosis is referenced to the National Institute of Neurological and Communicative Disorders and Stroke and Alzheimer’s Disease and Related Disorders Association (NINCDS/ADRDA) criteria (Dubois et al., 2007). In the present study, 86 participants were diagnosed with AD at T_3_ and 97 were diagnosed with AD from T_3_ to T_4_ (Figure 1).

### PET Imaging

Amyloid deposition and fluorodeoxyglucose (FDG) metabolism were visualized using 18F-florbetapir and 18F-FDG PET respectively. All image acquisition procedures were described in detail on the ADNI website.^2^ The mean florbetapir AV45 uptake within each region was calculated by co-registering the florbetapir scan to the corresponding MRI. 18F-florbetapir AV45-PET images were acquired in four frames of 5 min each, 50–70 min p.i. for 18F-florbetapir and 90–110 min p.i. for 18F-florbetaben. FDG-PET images (via averaging counts of angular, temporal, and posterior cingulate regions) were acquired on a PET template at the Montreal Neurological Institute with an isotropic resolution of 3 mm using FLIRT (Della Rosa et al., 2014).

### Brain MRI of hippocampus and white matter hyperintensities (WMH) measurement

All participants underwent high-resolution MRI of the brain scan at study entry (Jack et al., 2008). Structural brain images were obtained using a 1.5-T MRI system with T1-weighted MRI scans using a sagittal volumetric magnetization-prepared rapid acquisition gradient-echo sequence. The original MPRAGE (T1-weighted) structural volumetric MRI files were downloaded from UCSF.^3^ Here, the hippocampus was defined as the region of interest. This region was known to be affected by AD and their atrophy in AD has been previously validated via MRI studies (Pini et al., 2016; Katabathula et al., 2021). The WMH measurement approach has been described in detail on the ADNI site.^4^ Briefly, (1) non-brain tissues were removed from T1-weighted and FLAIR images; (2) the image pair was spatially aligned; and (3) artifacts were removed in MRI. Then, images were warped to a standard template space. At each location in the cerebral white matter, the prior probability of WMH occurrence and the FLAIR signal characteristics of WMHs were modeled. The prior information, along with the signal intensities of the FLAIR image, was used to identify WMH.

### Covariate measurements

The covariates include age, gender, years of education, *APOE*ε4 carrier status (number of *APOE* 4 alleles: 0, 1), clinical diagnosis (CN = 0, MCI = 1), intracranial volume (ICV), total white matter volume, SBP, and PP. Status of all covariates based on records at T_3_.

### Statistical analyses

Data were presented as mean (standard deviation, SD), median (interquartile range, IQR), or number (percentage, %) when appropriate. Chi-square tests (for categorical variables), Kruskal-Wallis test (for continuous variables with skewed distribution), and one-way ANOVA (for continuous variables with Gaussian distribution) were used to compare differences in participant characteristics among four SBPV groups (participants were divided into four groups according to SBPV quartiles). First, logistic regression model was conducted to determine the difference in SBPV between new-onset AD at T_3_ and their counterparts. Second, SBP trajectories prior to AD diagnosis were depicted for participants with incident AD and those who remained free of AD during 96-month follow-up (T_0_ to T_4_). The SBP data from the year of AD onset and from 1 to 8 years before AD onset were used to plot the SBP trajectory for participants with incident AD. The trajectory of SBP for those who remained non-demented was plotted using 8-year follow-up (T_0_ to T_4_) data. Third, the Cox proportional hazards models were conducted to assess roles of SBPV groups in predicting AD incidence (T_3_ to T_4_). Risk estimate was expressed as hazard ratios (HR) and corresponding 95% confidence interval (CI). We tested the proportional hazards assumption. The cumulative incidence curve for the cohort was measured using the Kaplan–Meier method and the curve difference was also calculated using the log-rank test.

Next, multiple linear regression models were used to examine the cross-sectional relationships of SBPV with cognitive function, and imaging endophenotypes (including AV45-PET, FDG-PET, hippocampus volume, and WMH) at T_3_. All dependent variables were checked for normal distribution and a transformation was done to approximate a normal distribution (Kolmogorov-Smirnov test *p*-value > 0.01) when the distribution is skewed. Models were visually checked for linearity of residuals, homogeneity of variances, and normality of residuals. There is no collinearity between the independent variables (variance inflation factor < 5). Finally, causal mediation analyses were performed to investigate the potential roles of AD imaging endophenotypes in modulating the relationship of SBPV with cognitive impairment based on MLR models (Baron and Kenny, 1986). The first equation analyzed the mediator (imaging endophenotypes) with the independent variable (SBPV). The second equation regressed the dependent variable (cognitive score) to the independent variable. The third equation regressed the dependent variable on both the independent and mediator variables. Mediation effects were established if the following criteria were simultaneously reached: 1) SBPV was significantly related to the mediator; 2) SBPV was significantly correlated to cognitive function; 3) the association of SBPV with cognition was attenuated when the mediator was added to the regression model. The indirect effect was estimated, with significance determined using 10,000 bootstrapped iterations with potential covariates adjusted. Additionally, the interaction terms of SBPV × *APOE* 4 (Sible and Nation, 2023; Ronnemaa et al., 2011) and SBPV × gender (Ernst et al., 2021) were added to the model to test the interactive effects. If the interaction analysis indicates significant results, further subgroup analyses based on *APOE* 4 genotype status or gender will be conducted.

The above-mentioned analyses were adjusted for age, gender, education levels, *APOE* 4 carrier status, and clinical diagnosis. ICV was added as a covariate when the dependent variable was hippocampus volume. When the dependent variable was WMH, total white matter volume was included as a covariate. SBP and PP at T_3_ were additionally adjusted for sensitivity analyses. R version 4.2.1 and GraphPad Prism 9.4.1 software were used for statistical analyses and figure preparation. The “glm”, “survival”, “survminer”, “ggplot2”, “nortest”, “car”, “performance”, “lm”, and “mediation” packages in R software were used to conduct the above analyses. Two-tailed tests were conducted, each with a significance level of 0.05.

## Results

### SBPV and AD risk

**FIGURE 2:**
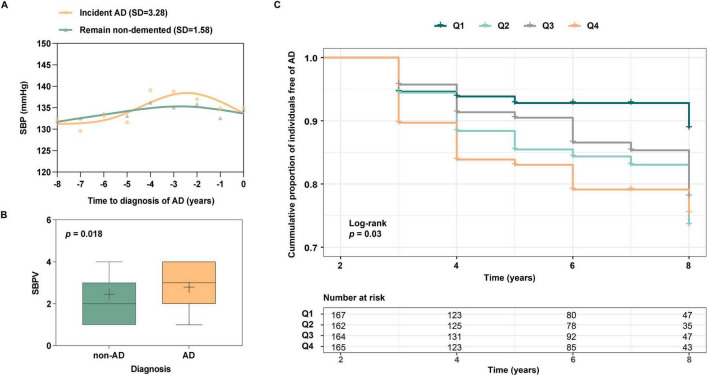
**(A)** SBP trajectories between those with incident AD vs those who remain non-demented for 8 years. The SBP trajectory for participants with incident AD was plotted using SBP data from the year of AD onset and from 1 to 8 years before AD onset. The trajectory of SBP for those who remained non-demented was plotted using 8-year follow-up (T_0_ to T_4_) data. Participants with incident AD have greater SBP fluctuations than those who remain non-demented; **(B)** Association of SBPV with AD patients and those who remain free of AD. Participants who incident AD at T_3_ were associated with greater SBPV compared to non-AD participants; **(C)** Association of SBPV with AD risk from T_3_ to T_4_. Participants with higher SBPV demonstrated a heightened risk of incident AD.

During the 96-month follow-up, 260 participants were diagnosed with AD, and 167 stayed non-demented at T_4_. Characteristics of these two groups at T_0_ are shown in Supplementary Table 1. Group differences were statistically significant in *APOE*ε4, clinical diagnosis, SBP, and PP (p < 0.05). SBP trajectory prior to AD diagnosis was plotted (Figure 2A). Compared to participants who remained non-demented (SD = 1.58), a higher SBPV (SD = 3.28) was observed before AD diagnosis. Next, the SBPV calculated from T_0_ to T_3_ was compared between 86 new-onset AD at T_3_ (n = 86, median age = 77.5, female = 37%) and 743 controls who were free of dementia at T_3_ (n = 743, median age = 77.0, female = 42%) (Supplementary Table 2). Group differences were observed in *APOE*ε4 percentage and SBPV (p < 0.05). In the fully-adjusted model, those incident AD exhibited higher SBPV (OR = 1.31, 95%CI: 1.06 to 1.63, *p* = 0.015, Figure 2B) compared with their controls. Finally, since T_3_, 658 non-demented participants were further followed up for 72 months (T_3_ to T_4_). Group differences were observed in age, SBP, and PP (Supplementary Table 3). The fully-adjusted Cox proportional hazards model after adjusting for age, gender, education, *APOE* 4, cognitive diagnosis, SBP, and PP at T_3_ showed that higher SBPV was associated with increased risk of incident AD (HR = 1.28, 95% CI: 1.05 to 1.56, *p* = 0.012, Figure 2C). In addition, a marginally significant interaction effect of SBPV × gender on AD risk was found (*p* = 0.078). Further subgroup analysis suggested that SBPV was associated with an increased risk of AD only in the female group (*p* = 0.005). No interaction effects were found for SBPV × *APOE* 4.

### Relationships of SBPV with cognition and imaging endophenotypes

At T_3_, 743 participants who were free of dementia were included. The median age of the participants was 77.0 years (IQR: 73.0-81.0) and 315 (42.40%) were female. The education attainment (median = 16 years) and *APOE* 4 percentage (34.19%) were relatively higher than general population (Wang et al., 2021). Participants in the high SBPV group tended to be older and exhibited poorer cognition (general cognition by ADAS13, memory, and executive function), higher levels of SBP and PP at T_3_, and higher burden of WMH. Group differences were observed in total white matter volume (*p* < 0.05) (Table 1).

**TABLE 1:** Characteristics of participants according to quartiles of SBPV at T_3_.

Characteristics	All participants	Q1^a^	Q2^a^	Q3^a^	Q4^a^	*P*-value*
N	743	187	185	185	186	
Age (years, median (IQR))	77.00 (73.00–81.00)	76.00 (72.00–80.00)	76.00 (73.00–80.00)	76.00 (73.00–81.00)	78.00 (74.00–82.00)	**0.004**
Sex (female, %)	315 (42.40)	80 (42.78)	81 (43.78)	69 (37.30)	85 (45.45)	0.398
Education (years, median (IQR))	16.00 (14.00–18.00)	16.00 (14.00–18.00)	16.00 (14.00–18.00)	16.00 (14.00–18.00)	16.00 (14.00–18.00)	0.535
*APOE*ε4 (yes, %)	254 (34.19)	62 (33.16)	66 (35.68)	65 (35.14)	61 (32.62)	0.918
Clinical diagnosis (MCI, %)	135 (18.17)	36 (19.25)	23 (12.43)	43 (23.24)	33 (17.74)	0.058
SBP (mmHg, mean ± SD)	133.91 ± 16.67	130.31 ± 12.71	132.65 ± 14.12	133.10 ± 15.10	139.59 ± 21.88	** < 0.001**
DBP (mmHg, median (IQR))	72.00 (66.00–80.00)	71.00 (66.00–79.00)	72.00 (66.00–80.00)	72.00 (65.00–80.00)	74.00 (66.00–81.25)	0.250
PP (mmHg, mean ± SD)	61.00 ± 15.21	58.17 ± 12.93	59.50 ± 12.95	60.82 ± 13.00	65.52 ± 19.85	** < 0.001**
ADAS-13 ^b^ (score, median (IQR))	11.00 (7.00–16.33)	10.00 (6.34–15.34)	10.00 (6.00–15.67)	11.67 (7.33–17)	12.00 (8.33–16.84)	**0.012**
ADNI-MEM ^c^ (score, mean ± SD)	0.68 ± 0.80	0.72 ± 0.79	0.79 ± 0.77	0.66 ± 0.86	0.56 ± 0.76	**0.037**
ADNI-EF ^c^ (score, mean ± SD)	0.61 ± 0.90	0.63 ± 0.88	0.73 ± 0.86	0.67 ± 0.90	0.42 ± 0.92	**0.004**
AV45-PET ^d^ (mmł, median (IQR))	0.76 (0.71–0.91)	0.74 (0.70–0.89)	0.76 (0.70–0.90)	0.75 (0.71–0.95)	0.78 (0.72–0.95)	0.182
FDG-PET ^e^ (mmł, median (IQR))	1.28 (1.23–1.32)	1.30 (1.26–1.33)	1.27 (1.23–1.32)	1.27 (1.23–1.32)	1.27 (1.23–1.32)	0.098
Hippocampus ^f^ (mmł, mean ± SD)	6904 ± 1066	6937 ± 1074	6942 ± 1010	6913 ± 1113	6813 ± 1072	0.770
ICV ^f^ (mmł, mean ± SD)	1534202 ± 15593	1541124 ± 146627	1526667 ± 175928	1532509 ± 155608	1536906 ± 143274	0.893
Total WMH ^g^ (mmł, median (IQR))	4.31 (2.09–10.75)	3.45 (1.85–7.92)	3.62 (2.00–7.32)	3.95 (2.19–9.54)	7.77 (3.01–14.10)	**0.002**
Total white matter volume ^g^ (mmł, mean ± SD)	465.00 ± 57.43	472.60 ± 58.81	466.10 ± 57.40	472.30 ± 53.08	448.10 ± 57.70	**0.012**

Values are mean ± standard deviation (SD), median (IQR (interquartile range)), or *n* (% of the group).

*Chi-square tests (for categorical variables), Kruskal-Wallis test (for non-normally distributed continuous variables), and one-way ANOVA (for normally distributed continuous variables) were used to compare characteristics. a, SBPV was calculated over T_0_ to T_3_ and divided into quartiles. Participants were divided into four groups according to SBPV quartiles. b, *N* = 738 c, *N* = 741 d, *N* = 329 e, *N* = 409 f, *N* = 510, g, *N* = 368. SBPV, systolic blood pressure variability; SD, standard deviation; IQR, interquartile range; *APOE*, apolipoprotein E gene; MCI, mild cognitive impairment; SBP, systolic blood pressure; DBP, diastolic blood pressure; PP, pulse pressure; ADAS-13, Alzheimer’s Disease Assessment Scale 13; ADNI, Alzheimer’s Disease Neuroimaging Initiative; EF, executive function; MEM, memory; PET, positron emission tomography; AV45, 18F florbetapir AV45 PET was used to estimate cerebral amyloid beta load; FDG, Fluorodeoxyglucose; MRI, magnetic resonance imaging; ICV, intracranial volume; WMH, white matter hyperintensity.

**FIGURE 3:**
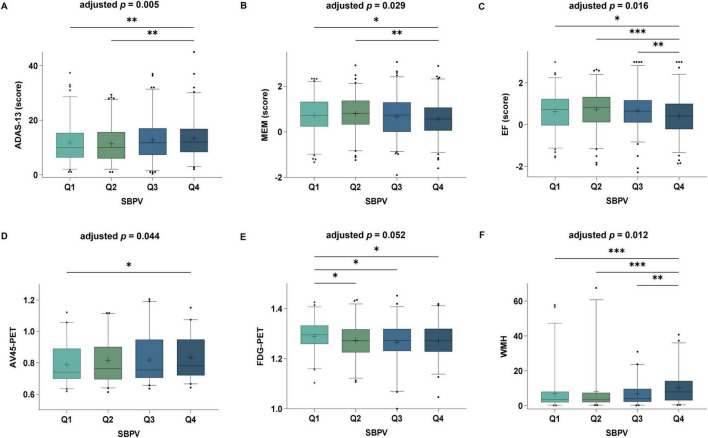
Associations of SBPV with cognition and AD imaging endophenotypes at T_3_. Participants with higher SBPV were associated with poorer global cognition **(A)**, memory **(B)**, executive function **(C)**, as well as higher amyloid load **(D)**, lower cerebral FluoroDeoxyGlucose (FDG) metabolism **(E)** and higher white matter hyperintensities (WMH) **(F)** compared to those with lower SBPV.

After adjusting for age, gender, education, *APOE* 4, and cognitive diagnosis at T_3_, we found that higher SBPV was associated with lower scores of general cognition by ADAS13 (β = 0.13, *p* = 0.005), memory function (β = −0.06, *p* = 0.029), and executive impairment (β = −0.07, *p* = 0.016). Moreover, higher SBPV was significantly associated with higher levels of brain *Aβ* burden (β = 0.02, *p* = 0.044), lower cerebral metabolism (β = −0.01, *p* = 0.052), and higher burden of WMH (β = 0.17, *p* = 0.012) (Figure 3). Sensitivity analyses after further adding PP and SBP in the model did not change the association of SBPV with general cognitive function (β = 0.11, *p* = 0.021) and WMH (β = 0.15, *p* = 0.032), but weakened that for memory (*p* = 0.083), executive function (*p* = 0.088), and cerebral metabolism (*p* = 0.070). Significant interaction effect of SBPV × gender on *Aβ* burden (*p* = 0.048) was found. Further subgroup analysis indicated that significant association between SBPV and *Aβ* burden was only in the male group (*p* = 0.005). No significant association was found between SBPV and hippocampus and no interaction effects were found for SBPV × *APOE* 4.

### Mediation analyses

**FIGURE 4:**
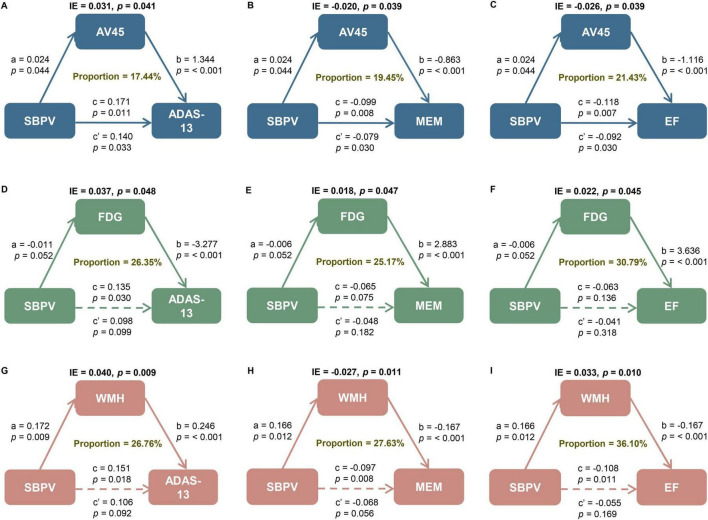
Mediation analyses with ADAS and cognitive domains as cognitive outcomes. The relationship of SBPV with cognitive measures, including **(A, D, G)** global cognition measured by ADAS as well as cognitive domain of **(B, E, H)** memory (MEM) and **(C, F, I)** executive function (EF) was mediated by **(A–C)** amyloid load, **(D–F)** FluoroDeoxyGlucose (FDG) metabolism, and **(G–I)** white matter hyperintensities (WMH). IE, indirect effect. SBPV, systolic blood pressure variability; AD, Alzheimer’s disease; SD, standard deviation; IQR, interquartile range; *APOE*, apolipoprotein E gene; MCI, mild cognitive impairment; SBP, systolic blood pressure; DBP, diastolic blood pressure; PP, pulse pressure; ADAS-13, Alzheimer’s Disease Assessment Scale 13; ADNI, Alzheimer’s Disease Neuroimaging Initiative; EF, executive function; MEM, memory; PET, positron emission tomography; AV45, 18F florbetapir; FDG, Fluorodeoxyglucose; WMH, white matter hyperintensities; MRI, magnetic resonance imaging; ICV, intracranial volume; *Aβ*,amyloid-beta; Q1-4, quartiles 1-4; T_0_, baseline; T_1_, month 6; T_2_, month 12; T_3_, month 24; T_4_, month 96.

Amyloid pathology by AV45 PET partially mediated the relationships of SBPV with general cognition (*p* = 0.041, proportion = 17.44%), memory function (*p* = 0.039, proportion = 19.45%), and executive function (*p* = 0.039, proportion = 21.43%). Brain metabolism by FDG PET mediated the relationships of SBPV with general cognition (*p* = 0.048, proportion = 26.35%), memory function (*p* = 0.047, proportion = 25.17%), and executive function (*p* = 0.045, proportion = 30.79%). The relationships of SBPV with general cognition (*p* = 0.009, proportion = 26.76%), memory function (*p* = 0.011, proportion = 27.63%), and executive function (*p* = 0.010, proportion = 36.10%) were also partially mediated by WMH (Figure 4).

## Discussion

We comprehensively investigated the associations of late-life SBPV with cognition, AD risk, and AD-associated neuroimaging markers. Our findings indicated that 1) late-life higher SBPV predicted poor cognition, elevated AD risk, increased amyloid burden, decreased brain metabolism, and increased WMH burden; and 2) *Aβ* pathology, brain metabolism, and WMH mediated the associations of SBPV with cognitive impairment. These findings supported that blood pressure management, especially maintaining a stable SBP trajectory could be a promising approach to preventing AD in older adults.

Our findings aligned with previous studies, which showed that higher SBPV predicted an increased risk of AD (Alperovitch et al., 2014; Mahinrad et al., 2023) among the elderly. These findings together highlighted the significance of maintaining stable SBP for AD prevention. Previous studies have reported that SBPV could be influenced by non-pharmaceutical factors [Mediterranean diet score (Lau et al., 2015), long sleep duration, and persistent insomnia (Nagai et al., 2013)] and antihypertensive medications [calcium-channel blockers and non-loop diuretic drugs (Webb et al., 2010; Webb and Rothwell, 2012)]. Additionally, we recently reported that the relationships of late-life BP with AD pathology and neurodegeneration could be modified by antihypertensive treatments (Guo et al., 2024). Further research is required to investigate whether such interventions could alter the associations of SBPV with AD. On the other hand, one study failed to reveal correlation between SBPV and AD risk (van Middelaar et al., 2018). The discrepant results could potentially be explained by that this study was conducted on an older population (aged 70 to 78 years). In addition, future studies are warranted to explore whether AD patients related to high SBPV represented a specific AD type.

Mediation analyses revealed potential pathways by which SBPV was involved in cognitive impairment via modulating *Aβ* pathology, brain metabolism, and WMH. Several possible biological mechanisms could explain these relationships. First, higher BPV may cause hemodynamic instability and induce shear stress on the vascular wall, possibly leading to microvascular damage. The cerebral microcirculatory dysfunction can damage the blood–brain barrier. Higher SBPV has been linked to arterial stiffness as well. Microvascular damage, arterial stiffness, and blood-brain barrier breakdown can further affect *Aβ* clearance (Gupta and Iadecola, 2015; Zlokovic, 2011; Tedla et al., 2017). In addition, BPV is an upstream determinant of artery remodeling. Hypoperfusion and hypoxia due to artery remodeling increase the secretion of proinflammatory cytokines and reactive oxygen species and induce microglia overactivation. The upregulation of the neuroinflammatory cascade and the reactive gliosis can enhance *Aβ* production (Nagai et al., 2017; Sible et al., 2021; Nagai et al., 2015; Nelson et al., 2014); Second, higher BPV may result in inconsistent perfusion and repeated episodes of tissue hypoxia-ischemia, leading to over-activation of the microglia (Rouch et al., 2020; Lattanzi et al., 2018b). There was a negative correlation between microglial activation and glucose metabolism (Fan et al., 2015). Reduced glucose metabolism can reflect poorer integrated synaptic activity (Rocher et al., 2003), which was associated with cognitive impairment related to neurodegenerative processes (Jack et al., 2010; Savva et al., 2009; Terry and Katzman, 2001); Third, arterial stiffness driven by higher BPV can further cause a “tsunami effect” towards the cerebral parenchyma, ultimately leading to WMH (Saji et al., 2016; Lattanzi et al., 2018a). Besides, higher BPV exposes the vessels to chronic stress. This may cause chronic hypoperfusion and impaired blood-brain barrier function, leading to WMH (Hilkens et al., 2024). WMH can disrupt brain white matter communication pathways associated with cognitive function (Wiseman et al., 2018; Vergoossen et al., 2021). Interestingly, previous studies identified the presence of a mediating effect of *Aβ* pathology on cognitive impairment associated with WMH (Ottoy et al., 2023), as well as a mediating effect of WMH on cognitive impairment related to *Aβ* pathology (Bernal et al., 2023). Future studies are warranted to explore whether WMH is involved in the pathway linking SBPV, *Aβ* pathology, and cognitive impairment.

The major strength of our study is that, to reduce the risk of reverse causality and immortal time bias, individuals who developed AD during the SBPV calculation period were excluded. The relationships of SBPV with AD risk and its neuroimaging markers were firstly comprehensively explored. Limitations should be acknowledged as well. First, this is an observational study. The results only reflect but cannot be equivalent to the causal relationships. Further investigations via in vivo or in vitro experiments should be conducted to confirm our findings about the impact of SBPV on metabolism of AD pathology. Randomized clinical trials are needed to test the efficiency of BPV management in preventing cognitive decline or dementia. Second, the competing risk due to cardiovascular disease mortality and confounding effects due to cardiovascular diseases and antihypertensive medications cannot be analyzed because of the limited data. According to previous publications, SBPV was significantly associated with cardiovascular disease mortality and events (Stevens et al., 2016). Antihypertensive medications might reduce the risk of dementia (Ding et al., 2020). Third, the generalizability of the results may be compromised by the fact that ADNI participants were highly educated volunteers. More large community-based longitudinal studies are warranted to validate these associations.

## Conclusion

To sum up, the present study indicated high SBPV could predict AD occurrence. The associations could be mediated by *Aβ* pathology, brain metabolism, and brain vessel health. These findings reinforced the value of maintaining stable SBP in preventing cognitive decline and incident AD. Further efforts are warranted to verify these findings in larger community-based studies.

## Data Availability

The raw data supporting the conclusions of this article will be made available by the authors, without undue reservation.

## Footnotes

1
http://adni.loni.usc.edu/

2
http://adni.loni.usc.edu/methods/documents/

3
https://ida.loni.usc.edu/pages/access/studyData.jsp

4
http://adni.loni.usc.edu

## References

[B1] Alperovitch A. Blachier M. Soumare A. Ritchie K. Dartigues J. Richard-Harston S. (2014). Blood pressure variability and risk of dementia in an elderly cohort, the three-city study. *Alzheimers Dement.* 10 S330–S337. 10.1016/j.jalz.2013.05.1777 23954028

[B2] Alzheimers Association Report (2022). 2022 Alzheimer’s disease facts and figures. *Alzheimers Dement.* 18 700–789. 10.1002/alz.12638 35289055

[B3] Baron R. Kenny D. (1986). The moderator-mediator variable distinction in social psychological research: Conceptual, strategic, and statistical considerations. *J. Pers. Soc. Psychol.* 51 1173–1182. 10.1037//0022-3514.51.6.1173 3806354

[B4] Bernal J. Schreiber S. Menze I. Ostendorf A. Pfister M. Geisendorfer J. (2023). Arterial hypertension and beta-amyloid accumulation have spatially overlapping effects on posterior white matter hyperintensity volume: A cross-sectional study. *Alzheimers Res. Ther.* 15:97. 10.1186/s13195-023-01243-4 37226207 PMC10207740

[B5] Crane P. Carle A. Gibbons L. Insel P. Mackin R. Gross A. (2012). Development and assessment of a composite score for memory in the Alzheimer’s Disease Neuroimaging Initiative (ADNI). *Brain Imaging Behav.* 6 502–516. 10.1007/s11682-012-9186-z 22782295 PMC3806057

[B6] de Heus R. Olde Rikkert M. Tully P. Lawlor B. Claassen J. Group N. (2019). Blood pressure variability and progression of clinical Alzheimer disease. *Hypertension* 74 1172–1180. 10.1161/HYPERTENSIONAHA.119.13664 31542965

[B7] de Heus R. Tzourio C. Lee E. Opozda M. Vincent A. Anstey K. (2021). Association between blood pressure variability with dementia and cognitive impairment: A systematic review and meta-analysis. *Hypertension* 78 1478–1489. 10.1161/HYPERTENSIONAHA.121.17797 34538105 PMC8516811

[B8] Della Rosa P. Cerami C. Gallivanone F. Prestia A. Caroli A. Castiglioni I. (2014). A standardized [18F]-FDG-PET template for spatial normalization in statistical parametric mapping of dementia. *Neuroinformatics* 12 575–593. 10.1007/s12021-014-9235-4 24952892

[B9] den Brok M. van Dalen J. Marcum Z. Busschers W. van Middelaar T. Hilkens N. (2023). Year-by-year blood pressure variability from midlife to death and lifetime dementia risk. *JAMA Netw. Open* 6:e2340249. 10.1001/jamanetworkopen.2023.40249 37902753 PMC10616718

[B10] Ding J. Davis-Plourde K. Sedaghat S. Tully P. Wang W. Phillips C. (2020). Antihypertensive medications and risk for incident dementia and Alzheimer’s disease: A meta-analysis of individual participant data from prospective cohort studies. *Lancet Neurol.* 19 61–70. 10.1016/S1474-4422(19)30393-X 31706889 PMC7391421

[B11] Dubois B. Feldman H. Jacova C. Dekosky S. Barberger-Gateau P. Cummings J. (2007). Research criteria for the diagnosis of Alzheimer’s disease: Revising the NINCDS-ADRDA criteria. *Lancet Neurol.* 6 734–746. 10.1016/S1474-4422(07)70178-3 17616482

[B12] Ernst M. Ryan J. Chowdhury E. Margolis K. Beilin L. Reid C. (2021). Long-term blood pressure variability and risk of cognitive decline and dementia among older adults. *J. Am. Heart Assoc.* 10:e019613. 10.1161/JAHA.120.019613 34176293 PMC8403315

[B13] Fan Z. Aman Y. Ahmed I. Chetelat G. Landeau B. Ray Chaudhuri K. (2015). Influence of microglial activation on neuronal function in Alzheimer’s and Parkinson’s disease dementia. *Alzheimers Dement.* 11:608–621 e607. 10.1016/j.jalz.2014.06.016 25239737

[B14] Gibbons L. Carle A. Mackin R. Harvey D. Mukherjee S. Insel P. (2012). Alzheimer’s disease neuroimaging I: A composite score for executive functioning, validated in Alzheimer’s disease neuroimaging initiative (ADNI) participants with baseline mild cognitive impairment. *Brain Imaging Behav.* 6 517–527. 10.1007/s11682-012-9176-1 22644789 PMC3684181

[B15] Guo Y. Tan C. Tan M. Tan L. Xu W. (2024). Anti-hypertensive drugs moderate the relationship of blood pressure with Alzheimer’s pathologies and neurodegenerative markers in non-demented hypertensive older adults. *J. Prev. Alzheimers Dis.* 11 672–683. 10.14283/jpad.2024.40 38706283

[B16] Gupta A. Iadecola C. (2015). Impaired Abeta clearance: A potential link between atherosclerosis and Alzheimer’s disease. *Front. Aging Neurosci.* 7:115. 10.3389/fnagi.2015.00115 26136682 PMC4468824

[B17] Hilkens N. de Leeuw F. Klijn C. Richard E. (2024). Blood pressure variability and white matter hyperintensities after ischemic stroke. *Cereb. Circ. Cogn. Behav.* 6:100205. 10.1016/j.cccb.2024.100205 38292015 PMC10827490

[B18] Hodson R. (2018). Alzheimer’s disease. *Nature* 559:S1. 10.1038/d41586-018-05717-6 30046078

[B19] Jack C. Bennett D. Blennow K. Carrillo M. Feldman H. Frisoni G. (2016). A/T/N: An unbiased descriptive classification scheme for Alzheimer disease biomarkers. *Neurology* 87 539–547. 10.1212/WNL.0000000000002923 27371494 PMC4970664

[B20] Jack C. Bernstein M. Fox N. Thompson P. Alexander G. Harvey D. (2008). The Alzheimer’s disease neuroimaging initiative (ADNI): MRI methods. *J. Magn. Reson. Imaging* 27 685–691. 10.1002/jmri.21049 18302232 PMC2544629

[B21] Jack C. Knopman D. Jagust W. Shaw L. Aisen P. Weiner M. (2010). Hypothetical model of dynamic biomarkers of the Alzheimer’s pathological cascade. *Lancet Neurol.* 9 119–128. 10.1016/S1474-4422(09)70299-6 20083042 PMC2819840

[B22] Katabathula S. Wang Q. Xu R. (2021). Predict Alzheimer’s disease using hippocampus MRI data: A lightweight 3D deep convolutional network model with visual and global shape representations. *Alzheimers Res. Ther.* 13:104. 10.1186/s13195-021-00837-0 34030743 PMC8147046

[B23] Lattanzi S. Vernieri F. Silvestrini M. (2018b). Blood pressure variability and neurocognitive functioning. *J. Clin. Hypertens.* 20 645–647. 10.1111/jch.13232 29466608 PMC8031143

[B24] Lattanzi S. Brigo F. Vernieri F. Silvestrini M. (2018a). Visit-to-visit variability in blood pressure and Alzheimer’s disease. *J. Clin. Hypertens.* 20 918–924. 10.1111/jch.13290 29693801 PMC8031352

[B25] Lau K. Wong Y. Chan Y. Li O. Lee P. Yuen G. (2015). Mediterranean-style diet is associated with reduced blood pressure variability and subsequent stroke risk in patients with coronary artery disease. *Am. J. Hypertens.* 28 501–507. 10.1093/ajh/hpu195 25352231

[B26] Li G. Rhew I. Shofer J. Kukull W. Breitner J. Peskind E. (2007). Age-varying association between blood pressure and risk of dementia in those aged 65 and older: A community-based prospective cohort study. *J. Am. Geriatr. Soc.* 55 1161–1167. 10.1111/j.1532-5415.2007.01233.x 17661953

[B27] Ma Y. Song A. Viswanathan A. Blacker D. Vernooij M. Hofman A. (2020a). Blood pressure variability and cerebral small vessel disease: A systematic review and meta-analysis of population-based cohorts. *Stroke* 51 82–89. 10.1161/STROKEAHA.119.026739 31771460 PMC7050788

[B28] Ma Y. Yilmaz P. Bos D. Blacker D. Viswanathan A. Ikram M. (2020b). Blood pressure variation and subclinical brain disease. *J. Am. Coll. Cardiol.* 75 2387–2399. 10.1016/j.jacc.2020.03.043 32408975 PMC9049233

[B29] Mahinrad S. Bennett D. Sorond F. Gorelick P. (2023). Blood pressure variability, dementia, and role of antihypertensive medications in older adults. *Alzheimers Dement.* 19 2966–2974. 10.1002/alz.12935 36656086 PMC10354219

[B30] Morris M. Scherr P. Hebert L. Glynn R. Bennett D. Evans D. (2001). Association of incident Alzheimer disease and blood pressure measured from 13 years before to 2 years after diagnosis in a large community study. *Arch. Neurol.* 58 1640–1646. 10.1001/archneur.58.10.1640 11594923

[B31] Mortamais M. Artero S. Ritchie K. (2014). White matter hyperintensities as early and independent predictors of Alzheimer’s disease risk. *J. Alzheimers Dis.* 42 S393–S400. 10.3233/JAD-141473 25261452

[B32] Nagai M. Dote K. Kato M. Sasaki S. Oda N. Kagawa E. (2017). Visit-to-visit blood pressure variability and Alzheimer’s disease: Links and risks. *J. Alzheimers Dis.* 59 515–526. 10.3233/JAD-161172 28598842

[B33] Nagai M. Hoshide S. Dote K. Kario K. (2015). Visit-to-visit blood pressure variability and dementia. *Geriatr. Gerontol. Int.* 15 26–33. 10.1111/ggi.12660 26671154

[B34] Nagai M. Hoshide S. Nishikawa M. Shimada K. Kario K. (2013). Sleep duration and insomnia in the elderly: Associations with blood pressure variability and carotid artery remodeling. *Am. J. Hypertens.* 26 981–989. 10.1093/ajh/hpt070 23723262

[B35] Nelson L. Gard P. Tabet N. (2014). Hypertension and inflammation in Alzheimer’s disease: Close partners in disease development and progression! *J. Alzheimers Dis.* 41 331–343. 10.3233/JAD-140024 24614908

[B36] Ottoy J. Ozzoude M. Zukotynski K. Adamo S. Scott C. Gaudet V. (2023). Vascular burden and cognition: Mediating roles of neurodegeneration and amyloid PET. *Alzheimers Dement.* 19 1503–1517. 10.1002/alz.12750 36047604

[B37] Ou Y. Tan C. Shen X. Xu W. Hou X. Dong Q. (2020). Blood pressure and risks of cognitive impairment and dementia: A systematic review and meta-analysis of 209 prospective studies. *Hypertension* 76 217–225. 10.1161/HYPERTENSIONAHA.120.14993 32450739

[B38] Ou Y. Xu W. Li J. Guo Y. Cui M. Chen K. (2019). FDG-PET as an independent biomarker for Alzheimer’s biological diagnosis: A longitudinal study. *Alzheimers Res. Ther.* 11:57. 10.1186/s13195-019-0512-1 31253185 PMC6599313

[B39] Petersen R. Aisen P. Beckett L. Donohue M. Gamst A. Harvey D. (2010). Alzheimer’s Disease Neuroimaging Initiative (ADNI): Clinical characterization. *Neurology* 74 201–209. 10.1212/WNL.0b013e3181cb3e25 20042704 PMC2809036

[B40] Pini L. Pievani M. Bocchetta M. Altomare D. Bosco P. Cavedo E. (2016). Brain atrophy in Alzheimer’s disease and aging. *Ageing Res. Rev.* 30 25–48. 10.1016/j.arr.2016.01.002 26827786

[B41] Qiu C. Kivipelto M. von Strauss E. (2009). Epidemiology of Alzheimer’s disease: Occurrence, determinants, and strategies toward intervention. *Dialogues Clin. Neurosci.* 11 111–128. 10.31887/DCNS.2009.11.2/cqiu 19585947 PMC3181909

[B42] Qiu C. von Strauss E. Fastbom J. Winblad B. Fratiglioni L. (2003). Low blood pressure and risk of dementia in the Kungsholmen project: A 6-year follow-up study. *Arch. Neurol.* 60 223–228. 10.1001/archneur.60.2.223 12580707

[B43] Rocher A. Chapon F. Blaizot X. Baron J. Chavoix C. (2003). Resting-state brain glucose utilization as measured by PET is directly related to regional synaptophysin levels: A study in baboons. *Neuroimage* 20 1894–1898. 10.1016/j.neuroimage.2003.07.002 14642499

[B44] Ronnemaa E. Zethelius B. Lannfelt L. Kilander L. (2011). Vascular risk factors and dementia: 40-year follow-up of a population-based cohort. *Dement. Geriatr. Cogn. Disord.* 31 460–466. 10.1159/000330020 21791923

[B45] Rouch L. Cestac P. Sallerin B. Piccoli M. Benattar-Zibi L. Bertin P. (2020). Visit-to-visit blood pressure variability is associated with cognitive decline and incident dementia: The S.AGES cohort. *Hypertension* 76 1280–1288. 10.1161/HYPERTENSIONAHA.119.14553 32862710

[B46] Ruitenberg A. Skoog I. Ott A. Aevarsson O. Witteman J. Lernfelt B. (2001). Blood pressure and risk of dementia: Results from the Rotterdam study and the Gothenburg H-70 Study. *Dement. Geriatr. Cogn. Disord.* 12 33–39. 10.1159/000051233 11125239

[B47] Saji N. Toba K. Sakurai T. (2016). Cerebral small vessel disease and arterial stiffness: Tsunami effect in the brain? *Pulse (Basel)* 3 182–189. 10.1159/000443614 27195239 PMC4865071

[B48] Savva G. Wharton S. Ince P. Forster G. Matthews F. Brayne C. (2009). Age, neuropathology, and dementia. *N. Engl. J. Med.* 360 2302–2309. 10.1056/NEJMoa0806142 19474427

[B49] Sible I. Nation D. (2023). Alzheimer’s disease neuroimaging I: Visit-to-visit blood pressure variability and cognitive decline in apolipoprotein varepsilon4 carriers versus apolipoprotein varepsilon3 homozygotes. *J. Alzheimers Dis.* 93 533–543. 10.3233/JAD-221103 37066910 PMC10852980

[B50] Sible I. Yew B. Dutt S. Bangen K. Li Y. Nation D. (2021). Alzheimer’s disease neuroimaging I: Visit-to-visit blood pressure variability and regional cerebral perfusion decline in older adults. *Neurobiol. Aging* 105 57–63. 10.1016/j.neurobiolaging.2021.04.009 34034215 PMC8979473

[B51] Stevens S. Wood S. Koshiaris C. Law K. Glasziou P. Stevens R. (2016). Blood pressure variability and cardiovascular disease: Systematic review and meta-analysis. *BMJ* 354:i4098. 10.1136/bmj.i4098 27511067 PMC4979357

[B52] Tedla Y. Yano Y. Carnethon M. Greenland P. (2017). Association between long-term blood pressure variability and 10-year progression in arterial stiffness: The multiethnic study of atherosclerosis. *Hypertension* 69 118–127. 10.1161/HYPERTENSIONAHA.116.08427 27849565 PMC5145729

[B53] Terry R. Katzman R. (2001). Life span and synapses: Will there be a primary senile dementia? *Neurobiol. Aging* 22:347–348; discussion 353–344. 10.1016/s0197-4580(00)00250-5 11378236

[B54] van Middelaar T. van Dalen J. van Gool W. van den Born B. van Vught L. (2018). Visit-to-visit blood pressure variability and the risk of dementia in older people. *J. Alzheimers Dis.* 62 727–735. 10.3233/JAD-170757 29480175

[B55] Vergoossen L. Jansen J. van Sloten T. Stehouwer C. Schaper N. Wesselius A. (2021). Interplay of white matter hyperintensities, cerebral networks, and cognitive function in an adult population: Diffusion-tensor imaging in the maastricht study. *Radiology* 298 384–392. 10.1148/radiol.2021202634 33350892

[B56] Wang Y. Ge Y. Tan C. Cao X. Tan L. Xu W. (2021). The proportion of APOE4 carriers among non-demented individuals: A pooled analysis of 389,000 community-dwellers. *J. Alzheimers Dis.* 81 1331–1339. 10.3233/JAD-201606 33935087

[B57] Webb A. Rothwell P. (2012). The effect of antihypertensive treatment on headache and blood pressure variability in randomized controlled trials: A systematic review. *J. Neurol.* 259 1781–1787. 10.1007/s00415-012-6449-y 22354262

[B58] Webb A. Fischer U. Mehta Z. Rothwell P. (2010). Effects of antihypertensive-drug class on interindividual variation in blood pressure and risk of stroke: A systematic review and meta-analysis. *Lancet* 375 906–915. 10.1016/S0140-6736(10)60235-8 20226989

[B59] Weiner M. Aisen P. Jack C. Jr. Jagust W. Trojanowski J. Shaw L. (2010). The Alzheimer’s disease neuroimaging initiative: Progress report and future plans. *Alzheimers Dement.* 6:202–211.e207. 10.1016/j.jalz.2010.03.007 20451868 PMC2927112

[B60] Wiseman S. Booth T. Ritchie S. Cox S. Munoz Maniega S. Valdes Hernandez M. (2018). Cognitive abilities, brain white matter hyperintensity volume, and structural network connectivity in older age. *Hum. Brain Mapp.* 39 622–632. 10.1002/hbm.23857 29139161 PMC5813175

[B61] Xu W. Tan L. Wang H. Jiang T. Tan M. Tan L. (2015). Meta-analysis of modifiable risk factors for Alzheimer’s disease. *J. Neurol. Neurosurg. Psychiatry* 86 1299–1306. 10.1136/jnnp-2015-310548 26294005

[B62] Yu J. Xu W. Tan C. Andrieu S. Suckling J. Evangelou E. (2020). Evidence-based prevention of Alzheimer’s disease: Systematic review and meta-analysis of 243 observational prospective studies and 153 randomised controlled trials. *J. Neurol. Neurosurg. Psychiatry* 91 1201–1209. 10.1136/jnnp-2019-321913 32690803 PMC7569385

[B63] Zlokovic B. (2011). Neurovascular pathways to neurodegeneration in Alzheimer’s disease and other disorders. *Nat. Rev. Neurosci.* 12 723–738. 10.1038/nrn3114 22048062 PMC4036520

