# Supplementary material for: Systolic blood pressure variability in late-life predicts cognitive trajectory and risk of Alzheimer’s disease
Source: Front Aging Neurosci. 2024 Oct 3;16:1448034. doi: 10.3389/fnagi.2024.1448034 (PMC11483855; doi:10.3389/fnagi.2024.1448034)
Supplement: Supplementary file 1 [file Data_Sheet_1.pdf]

CONTENT

**TITLE**	**Page**
**Supplementary Table S1: Characteristics of participants according to SBP trajectory.**	2
**Supplementary Table S2: Characteristics of participants according to AD diagnosis at T_3.**	3
**Supplementary Table S3: Characteristics of participants according to SBPV quartiles at T_3.**	4

Supplementary Table S1: Characteristics of participants according to SBP trajectory.

**Characteristics** [a]	**Incident AD** [b]	**Non-demented** [c]	***P*-value***
N	260	167	
Age (years, median (IQR))	75.00 (71.25-80.00)	74.00 (71.00-78.00)	0.080
Sex (female, %)	100 (38.46)	65 (38.92)	0.924
Education (years, median (IQR))	16.00 (14.00-18.00)	16.00 (14.00-18.00)	0.279
APOE ε4 (yes, %)	166 (63.85)	46 (27.54)	**<0.001**
Clinical diagnosis (MCI, %)	240 (92.31)	68 (40.72)	**<0.001**
SBP (mmHg, mean ± SD)	135.50 ± 17.14	131.80 ± 15.49	**0.026**
DBP (mmHg, median (IQR))	72.5 (67.00-80.00)	73.00 (67.00-80.00)	0.691
PP (mmHg, mean ± SD)	62.68 ± 14.41	58.44 ± 14.10	**0.003**

Values are mean ± standard deviation (SD), median (IQR (interquartile range)), or n (% of the group).

*Chi-square tests (for categorical variables), Mann-Whitney test (for non-normally distributed continuous variables), and t-test (for normally distributed continuous variables) were used to compare characteristics.

a, characteristics of participants are based on the records at T_0

b, participants who developed AD during the 8-year follow-up

c, participants who still remained non-demented at the end (T_4) of 8-year follow-up

Abbreviations: AD, Alzheimer's disease; IQR, interquartile range; SD, standard deviation; *APOE,* apolipoprotein E gene; MMSE, Minimum Mental State Examination; SBP, systolic blood pressure; DBP, diastolic blood pressure; PP, pulse pressure

Supplementary Table S2: Characteristics of participants according to AD diagnosis at T_3.

Characteristics [a]	All participants [b]	AD participants [c]	Controls	P-value*
N	829	86	743	
Age (years, median (IQR))	77.00 (73.00-81.00)	77.50 (73.00-81.25)	77.00 (73.00-81.00)	0.644
Sex (female, %)	347 (41.86)	32 (37.21)	315 (42.40)	0.356
Education (years, median (IQR))	16.00 (14.00-18.00)	16.00 (14.00-18.00)	16.00 (14.00-18.00)	0.929
APOE ε4 (yes, %)	314 (37.88)	60 (69.77)	254 (34.19)	**<0.001**
SBP (mmHg, median (IQR))	133.00 (122.00-144.00)	134.00 (120.80-149.00)	133.00 (123.00-144.00)	0.821
DBP (mmHg, median (IQR))	72.00 (66.00-80.00)	73.00 (67.00-80.00)	72.00 (66.00-80.00)	0.709
PP (mmHg, median (IQR))	60.00 (50.00-70.00)	59.50 (49.50-70.75)	60.00 (50.00-70.00)	0.930
SBPV Q1 [d] (n, %)	211 (25.45)	14 (16.28)	197 (26.51)	**0.039**
SBPV Q2 [d] (n, %)	206 (24.85)	19 (22.09)	187 (25.17)	0.532
SBPV Q3 [d] (n, %)	205 (24.73)	24 (27.91)	181 (24.36)	0.471
SBPV Q4 [d] (n, %)	207 (24.97)	29 (33.72)	178 (23.96)	**0.048**

Values are mean ± standard deviation (SD), median (IQR (interquartile range)), or n (% of the group).

*Chi-square tests (for categorical variables) and Mann-Whitney test (for non-normally distributed continuous variables) were used to compare characteristics.

a, characteristics of participants are based on the records at T_3

b, participants for case-control study

c, participants who were new onset AD at T_3

d, SBPV was defined as the within-individual SD of four SBP measurements (T_0, T_1, T_2, and T_3) and divided into four quartiles (Q1, Q2, Q3, and Q4).

Abbreviations: AD, Alzheimer's disease; SD, standard deviation; IQR, interquartile range; *APOE,* apolipoprotein E gene; MMSE, Minimum Mental State Examination; SBP, systolic blood pressure; DBP, diastolic blood pressure; PP, pulse pressure; SBPV, systolic blood pressure variability; Q1, quartile 1; Q2, quartile 2; Q3, quartile 3; Q4, quartile 4.

Supplementary Table S3: Characteristics of participants according to SBPV quartiles at T_3.

Characteristics [a]	All participants [b]	Q1 [c]	Q2 [c]	Q3 [c]	Q4 [c]	*P*-value*
N	658	167	162	164	165	
Age (years, median (IQR))	76.00 (73.00-81.00)	75.00 (72.00-80.00)	76.00 (73.00-80.25)	76.00 (73.00-81.00)	78.00 (73.00-82.00)	**0.029**
Sex (female, %)	285 (43.31)	69 (41.32)	73 (45.06)	70 (42.68)	73 (44.24)	0.906
Education (years, median (IQR))	16.00 (14.00-18.00)	16.00 (14.00-18.00)	16.00 (14.00-18.00)	16.00 (14.25-18.00)	16.00 (14.00-18.00)	0.529
APOE ε4 (yes, %)	222 (33.74)	56 (33.53)	52 (32.10)	56 (34.15)	58 (35.15)	0.949
Clinical diagnosis (MCI, %)	110 (16.72)	28 (16.77)	29 (17.90)	27 (16.46)	26 (15.76)	0.964
SBP (mmHg, mean ± SD)	133.80 ± 16.42	130.50 ± 13.09	132.90 ± 14.29	132.60 ± 14.60	139.20 ± 21.25	**<0.001**
DBP (mmHg, median (IQR))	72.00 (66.00-80.00)	70.00 (66.00-78.00)	73.00 (66.00-80.00)	72.00 (65.00-80.00)	74.00 (67.00-82.00)	0.070
PP (mmHg, mean ± SD)	60.72 ± 14.77	58.47 ± 13.11	59.38 ± 13.27	60.39 ± 12.40	64.64 ± 18.76	**0.006**

Values are mean ± standard deviation (SD), median (IQR(interquartile range)), or n (% of the group).

* Chi-square tests (for categorical variables), Kruskal-Wallis test (for non-normally distributed continuous variables), and one-way ANOVA (for normally distributed continuous variables) were used to compare characteristics.

a, characteristics of participants are based on the records at T_3

b, participants for Cox proportional hazard model.

c, SBPV was calculated over T_0 to T_3 and divided into quartiles. Participants were divided into four groups according to SBPV quartiles.

Abbreviations: SBPV, systolic blood pressure variability; IQR, interquartile range; SD, standard deviation; *APOE,* apolipoprotein E gene; MCI, mild cognitive impairment; SBP, systolic blood pressure; DBP, diastolic blood pressure; PP, pulse pressure
